# Performance of OCT-Based Artificial Intelligence Models for Detecting and Predicting Glaucoma Progression: A Systematic Review

**DOI:** 10.3390/jcm15155976

**Published:** 2026-07-31

**Authors:** Rayan Abdullah J. Alzahrani, Abdulmalek W. Alhithlool, Ali Saleh Alsudais, Amal Salem AlHarbi, Shahad Fouad Alyousif, Mohammed Naji Almutairi, Khulood Sultan Alharbi, Atheer Mohammed Almalki, Basmah Abdullah Alshehri, Amnah Ali Alkhawajah, Sultan S. Aldrees

**Affiliations:** 1 College of Medicine, Al-Baha University, Al-Baha 65779, Saudi Arabia; 2 College of Medicine, King Faisal University, Al-Ahsa 31982, Saudi Arabia; 3 College of Medicine, King Saud bin Abdulaziz University for Health Sciences, Jeddah 21423, Saudi Arabia; 4 Department of Ophthalmology, Dammam Medical Complex, Dammam 31196, Saudi Arabia; 5 College of Medicine, King Saud bin Abdulaziz University for Health Sciences, Riyadh 11481, Saudi Arabia; 6 College of Medicine, University of Jeddah, Jeddah 21589, Saudi Arabia; 7 College of Medicine, King Abdulaziz University, Jeddah 21589, Saudi Arabia; 8 College of Medicine, King Khalid University, Abha 61421, Saudi Arabia; 9 Research Excellence Center in Ophthalmology and Visual Sciences, Department of Ophthalmology, College of Medicine, King Saud University, Riyadh 11451, Saudi Arabia; 10 King Saud University Medical City, Riyadh 11362, Saudi Arabia

**Keywords:** artificial intelligence, deep learning, glaucoma, machine learning, optical coherence tomography

## Abstract

**Background/Objectives**: Glaucoma is a leading cause of irreversible blindness globally and must be detected early to preserve vision. Standard methods, such as automated perimetry and optical coherence tomography (OCT), are limited in their ability to detect early changes. Therefore, this systematic review evaluates the diagnostic and predictive performance of artificial intelligence (AI)-driven OCT-based models, including those based on deep learning, machine learning, and transformer architectures, and contextualizes their performance against the recognized limitations of standard automated perimetry and conventional OCT trend analysis. **Methods**: The PubMed, Ovid, and Google Scholar databases were comprehensively searched to identify relevant published research in English. Screening of observational, cohort, prospective, retrospective and diagnostic accuracy studies, as well as clinical trials, identified 13 studies. Their key outcomes, including sensitivity, specificity, and area under the curve (AUC), were extracted for synthesis. **Results**: AI-driven OCT-based models generally reported high diagnostic performance, with mean sensitivities and specificities exceeding 80%, as well as AUC values generally above 0.85. AI-driven OCT-based models were reported to detect glaucoma progression as early as 9 months before standard methods. **Conclusions**: The findings suggest that AI-driven OCT-based models may improve glaucoma detection by identifying subtle structural and functional changes earlier, which may help inform earlier intervention decisions. However, the majority of included studies relied on internal validation only, and prospective multicenter studies with external validation are required before AI-driven OCT-based models can be reliably implemented in routine glaucoma care.

## 1. Introduction

Glaucoma is a chronic, progressive optic neuropathy that leads to irreversible vision loss. It is the leading cause of irreversible blindness worldwide, and its prevalence is projected to increase sharply, reaching approximately 111.8 million by 2040 [1,2]. Despite advances in detection and management, nearly half of glaucoma cases remain undetected [3] until substantial visual field (VF) loss has occurred, primarily because the early stages are asymptomatic [4]. Clinically, reducing intraocular pressure (IOP) remains the only proven treatment to slow glaucoma progression [5]. Therefore, detecting glaucoma progression early is essential to prevent irreversible vision loss. However, existing monitoring methods exhibit notable limitations in sensitivity, reproducibility, and consistency, particularly in detecting early glaucoma progression.

Glaucoma progression can be monitored by progressive functional deterioration on serial VF testing or progressive structural changes involving the optic nerve head and retinal nerve fiber layer (RNFL) [6]. Although VF testing is often used to detect functional glaucoma progression [7], it has several well-known limitations: it is time-consuming, has high test–retest variability, and typically requires multiple examinations to confirm progression [8,9]. As a subjective psychophysical examination, its reliability largely depends on patient cooperation and consistent performance. Learning effects may lead to apparent improvement with repeated testing, while cognitive impairment or language barriers can compromise understanding of instructions. Fluctuations in attention, motivation, or physical ability can also further reduce its reliability [10].

Optical coherence tomography (OCT) has become a cornerstone of glaucoma care, providing high-resolution, quantitative imaging of the optic nerve head and retinal layers. It enables objective measurement of the thickness of the RNFL and ganglion cell layer, which reflect glaucomatous damage [11]. Although in principle, serial OCT scans can document structural changes over time, in practice, assessing glaucoma progression remains challenging due to several limitations. For instance, OCT measurements can be affected by image artifacts and reliance on normative databases, which may result in false positives or negatives, leading to so-called “red-green” disease in some patients, where red indicates values outside the normative database and green indicates values within it [12]. The quality of the OCT input itself is also an active area of development, and recent advances in OCT image reconstruction—such as deconvolution methods that reduce ringing artifacts and improve image clarity—may in turn improve the reliability of the structural measurements on which AI models depend [12,13]. These challenges highlight the need for more sensitive and standardized tools.

Artificial intelligence (AI) has significantly advanced glaucoma care, particularly in screening, diagnosis, and detection of glaucoma progression [14]. Among the various AI algorithms, machine learning (ML), including deep learning (DL), has been increasingly applied in clinical science, with notable applications in glaucoma for detecting both structural and functional damage [15]. However, despite these promising developments, important gaps remain, especially regarding the use of AI to detect structural glaucoma progression using OCT.

Chen et al. noted that until recently, most AI models for detecting glaucoma progression have been based on VF data, often classifying patients as “progressors” or “non-progressors” [1]. In contrast, OCT-based AI models to detect glaucoma progression are only beginning to emerge, although several recent studies support their potential. For instance, Mariottoni et al. developed a convolutional neural network (CNN) that analyzed paired RNFL thickness profiles, which achieved an area under the receiver operating characteristic curve (AUC) of 0.938 for detecting structural glaucoma progression, significantly outperforming conventional trend analysis methods [16]. Furthermore, Huang et al. developed a deep learning model using macular OCT imaging that demonstrated high accuracy in detecting clinically significant glaucoma progression and predicting future progression, achieving AUC values of approximately 0.90 and 0.85, respectively [17].

More broadly, studies published between 2022 and 2024 suggest a shift toward architectures designed to exploit serial rather than single-visit OCT data, including vision transformers with self-supervised pretraining, gated transformer networks, CNN-LSTM temporal models, and pseudo-supervised multimodal networks [17,18,19,20,21].

A recent systematic review and meta-analysis by Ling et al. (2025) [22] evaluated deep learning algorithms for glaucoma diagnosis and progression prediction using both fundus photography and OCT imaging, reporting pooled area under the receiver operating characteristic curve (AUROC) values of 0.90 (95% CI: 0.88–0.92) for fundus- and 0.86 (95% CI: 0.83–0.90) for OCT-based diagnosis, with less robust performance for progression prediction and higher accuracy on internal compared to external validation datasets [22]. The present review is narrower in focus and complementary in aim: it is restricted to OCT-based AI models evaluated for detecting and predicting glaucoma progression, enabling closer examination of OCT input modalities, longitudinal modeling strategies, and the validation gap relevant to progression monitoring.

While AI has been widely applied to diagnosing glaucoma, there is a clear gap in its use for monitoring glaucoma progression over time, particularly using OCT data. Therefore, this systematic review aims to assess the diagnostic and predictive performance of AI-driven OCT-based models for detecting and predicting glaucoma progression and to contextualize their performance against the recognized limitations of standard automated perimetry and conventional OCT trend analysis.

## 2. Materials and Methods

### 2.1. Literature Review and Study Design

This systematic review was planned and executed in accordance with the *Cochrane Handbook for Systematic Reviews of Interventions*, and its findings are presented in accordance with the Preferred Reporting Items for Systematic Reviews and Meta-Analyses (PRISMA) guidelines [23]. The PRISMA checklist is available in Supplementary Materials. The review protocol was prospectively registered in the PROSPERO database (registration number: CRD420251039683). As this review retrospectively examined data from published studies, it did not require ethical approval. Nonetheless, it adhered to the principles outlined in the Declaration of Helsinki.

The registered protocol specified additional secondary outcomes, including inter-observer agreement, false-positive and false-negative rates, clinician confidence scores, and cost-effectiveness. These outcomes were reported in too few studies and with inconsistent definitions to permit reliable synthesis; accordingly, the analysis focused on primary diagnostic performance and temporal detection metrics, which were consistently reported across the included studies. A planned reporting-bias assessment was not conducted, as the descriptive synthesis did not generate pooled estimates required for such methods. All other aspects of the registered protocol were followed as planned.

### 2.2. Search Strategy

The online PubMed, Ovid, and Google Scholar databases were comprehensively and systematically searched on 11 November 2024 with no restrictions on publication date. No additional filters were applied to the database searches; language restriction to English was applied during the screening phase. The search strategy was tailored to each database using relevant keywords and Boolean operators. For the PubMed database, the search query was: (artificial intelligence OR machine learning OR deep learning) AND glaucoma progression. For the Ovid database, the search query was: ((AI or Artificial Intelligence) and Glaucoma).af. and Progression.ab. For the Google Scholar database, the search query was: “artificial intelligence glaucoma progression” OR “machine learning glaucoma” OR “deep learning glaucoma.” In addition to the database searches, the reference lists of all included studies were manually screened to identify additional relevant articles. One additional eligible study was identified through forward citation searching of the included studies on 1 June 2025 and was subsequently included. Although the core search strings combined artificial intelligence and glaucoma progression terms rather than device-specific keywords, the eligibility criteria required an OCT-based input, so optical coherence tomography modalities (including RNFL, macular, and optic nerve head OCT) were applied at the screening stage; this ensured that OCT-based studies were retained without narrowing the initial retrieval. Studies using terms such as “optical coherence tomography,” “RNFL,” “retinal nerve fiber layer,” and “macular OCT” were thereby captured.

### 2.3. Study Selection

All retrieved records were entered into Zotero (Version 8), and any duplicates were removed [24]. Initially, four reviewers independently evaluated the titles and abstracts to determine study eligibility; if they lacked sufficient information, the full-text articles were screened. Any discrepancies were resolved by a fifth independent reviewer, who adjudicated disagreements and verified the final list of included studies. The inclusion criteria were as follows: original research articles published in English reporting randomized controlled trials, prospective cohort studies, or case–control studies that employed AI algorithms to analyze OCT data and reported at least one outcome related to glaucoma progression detection; no restriction on publication year was applied. The exclusion criteria were as follows: not published in English, not reporting original research (e.g., editorials, narrative reviews, and systematic reviews) or reporting studies with inappropriate designs (e.g., meta-analyses, economic analyses, or involving animals or cadavers), or did not report relevant outcomes of interest.

### 2.4. Screening and Data Extraction

Four reviewers independently screened the full text of the included articles using the Rayyan AI-powered web-based systematic review management tool. They extracted the following data independently using a standardized form: study characteristics (first author, year of publication, country of origin, study design, and population demographics), cohort characteristics (glaucoma type, mean age, and sample size), technical model architecture (type of DL or ML algorithm, validation methodology [internal vs. external], and imaging/clinical data types), performance metrics (sensitivity [%], specificity [%], accuracy [%], and AUC for both AI models and clinicians, where available), and temporal metrics (time to detection of progression [months] and early detection improvement rates [%]). Disagreements were addressed through discussion or by consulting a fifth reviewer. Then, two reviewers independently verified all extracted data. When specific performance metrics were not directly reported, values were derived from available data where feasible (e.g., sensitivity and specificity calculated from reported confusion matrices); otherwise, the metric was recorded as not reported.

### 2.5. Risk of Bias Assessment

The methodologic quality of the included studies was evaluated using the Quality Assessment of Diagnostic Accuracy Studies–Version 2 (QUADAS-2) tool, which assesses four domains—“patient selection,” “index test,” “reference standard,” and “flow and timing”—alongside judgments on bias and applicability [25]. Each study was evaluated for risk of bias, and the initial three domains were examined concerning their applicability. Each item is rated as “yes,” “no,” or “unclear.” A “yes” response indicates a low risk of bias, whereas “no” or “unclear” indicates a high or unclear risk of bias. Data management, risk-of-bias judgments, and the generation of the quality assessment plots were performed using the web-based Review Manager tool (RevMan, web version; The Cochrane Collaboration, London, UK) [26].

### 2.6. Data Synthesis

The diagnostic and predictive performance of the AI-driven OCT-based models was summarized descriptively. Due to significant heterogeneity in AI model architectures, reference standards for defining glaucoma progression, and reported outcome measures across the included studies, a formal meta-analysis was not performed; accordingly, subgroup analyses, meta-regression, and sensitivity analyses were also not applicable. Performance metrics were summarized as ranges and means with standard deviations across the included studies. Formal assessment of reporting bias (e.g., funnel plot analysis) was not conducted, as these methods require pooled estimates derived from comparable studies reporting the same effect measures. A formal certainty-of-evidence assessment was not applied; instead, the overall methodological quality of the evidence was evaluated through the QUADAS-2 risk-of-bias assessment presented in the Results (Section 3).

## 3. Results

### 3.1. Characteristics of the Included Studies

**Figure 1:**
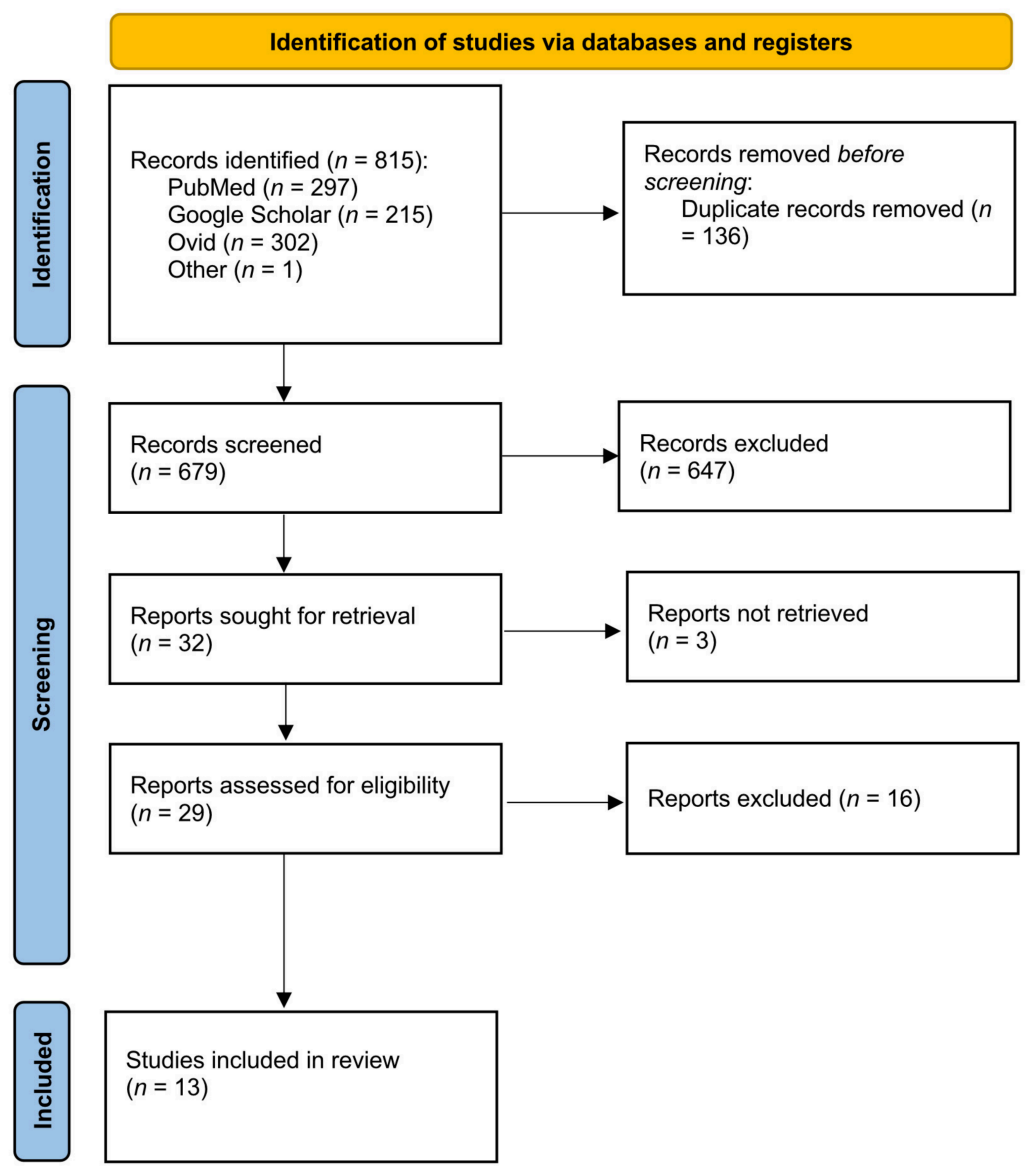
PRISMA flow diagram of the study selection process.

The database search and study selection process is shown as a PRISMA flow diagram in Figure 1. A total of 13 studies published between 2018 and 2024 met the eligibility criteria (Table 1). These studies encompassed over 14,000 eyes across diverse populations, including primary open-angle glaucoma, normal-tension glaucoma, ocular hypertension, and mixed or secondary glaucomas. Most studies were conducted in the USA (*n* = 10, 76.9%), with additional contributions from Asia (*n* = 1, 7.7%), Europe (*n* = 2, 15.4%), and South America (*n* = 1, 7.7%). Two studies were conducted across more than one country (USA and Brazil; UK and Romania), so country attributions exceed the total number of studies. Most studies employed a retrospective cohort design (11, 84.6%), while two employed a prospective design [27,28]. OCT was the dominant imaging modality across all included studies, often supplemented with VF and IOP assessments. Twelve studies (92.3%) used internal validation (e.g., 5-fold, 10-fold, or hold-out sets), and only one study (7.7%) [29] employed both internal and external validation using a distinct OCT dataset.

This near-universal reliance on internal validation, combined with the predominance of single-center cohorts, may limit the generalizability of the reported diagnostic and predictive performance. Estimates derived from a single institution’s OCT acquisition protocols, patient demographics, and reference-standard definitions may not transfer directly to other clinical populations or device platforms.

The ML algorithms employed varied substantially. Earlier studies used traditional ML methods (random forest, support vector machine, and logistic regression), whereas later studies adopted DL algorithms, including CNNs, long short-term memory (LSTM)-based temporal models, and transformer architectures [18,19]. These models increasingly incorporated longitudinal OCT data to capture patterns of structural glaucoma progression. Across studies, participants’ mean age ranged from 54 to 71 years, and dataset sizes ranged from 45 to over 4000 eyes. The overall methodological rigor was high, but external validation and multicenter reproducibility remain limited.

Across the included studies, OCT inputs broadly fell into four categories: RNFL-based structural measurements, map-based OCT representations, direct OCT B-scan inputs, and longitudinal time-series OCT.

### 3.2. Risk of Bias in Included Studies

**Figure 2:**
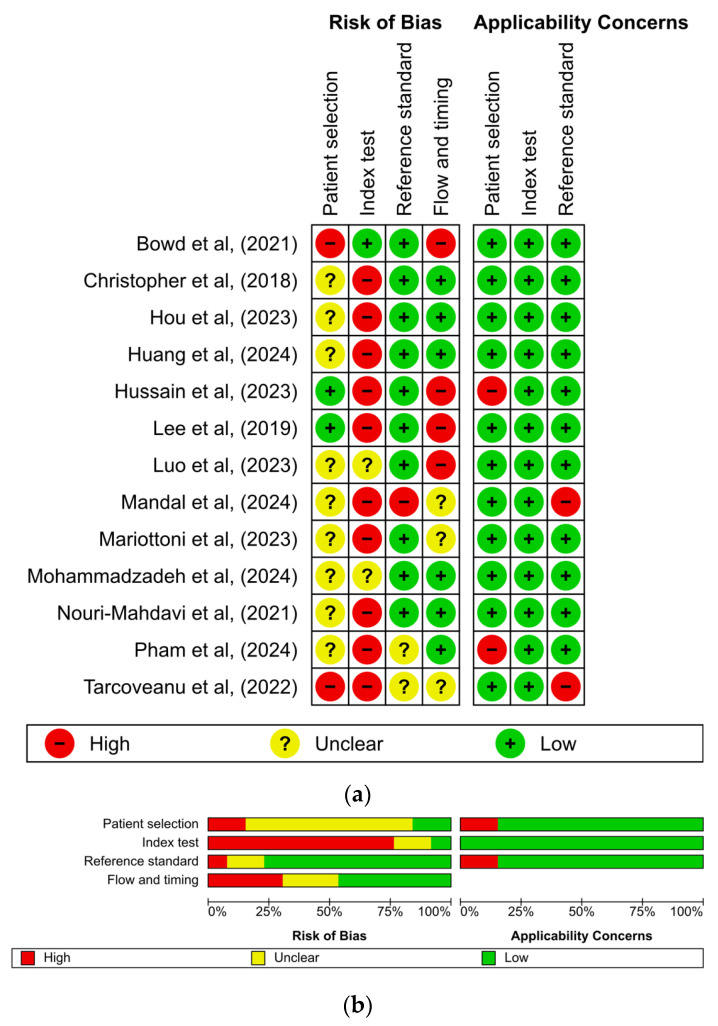
(**a**) Summary of the risk of bias and applicability concerns for each included study across the QUADAS-2 domains. (**b**) Graph summarizing the risk of bias and applicability concerns across the included studies in each QUADAS-2 domain. Included studies: Bowd et al. (2021) [27], Christopher et al. (2018) [30], Hou et al. (2023) [21], Huang et al. (2024) [17], Hussain et al. (2023) [19], Lee et al. (2019) [15], Luo et al. (2023) [20], Mandal et al. (2024) [18], Mariottoni et al. (2023) [16], Mohammadzadeh et al. (2024) [29], Nouri-Mahdavi et al. (2021) [28], Pham et al. (2024) [32], and Tarcoveanu et al. (2022) [31].

Across the 13 included studies, the risk of bias varied by domain (Figure 2). In the patient selection domain, 9 (69.2%) had an unclear risk of bias, 2 (15.4%) had a low risk of bias, and 2 (15.4%) had a high risk of bias. In the index test domain, 10 (76.9%) had a high risk of bias, 2 (15.4%) had an unclear risk of bias, and 1 (7.7%) had a low risk of bias. In the reference standard domain, 10 (76.9%) had a low risk of bias, 2 (15.4%) had an unclear risk of bias, and 1 (7.7%) had a high risk of bias. In the flow and timing domain, 6 (46.2%) had a low risk of bias, 4 (30.8%) had a high risk of bias, and 3 (23.1%) had an unclear risk of bias.

Overall, there were minimal concerns about the applicability of the studies included in this systematic review. High applicability concerns were noted in only two studies within the patient selection domain and two in the reference standard domain. All 13 included studies had low applicability concerns regarding the index test.

### 3.3. Diagnostic Performance of AI Models Compared with Clinicians

The performance metrics for the AI-driven models and clinician comparators are summarized in Table 2. Across studies reporting these outcomes, the sensitivity of AI-driven models ranged from 66% to 100% (mean = 83.6 ± 10.8%), and their specificity ranged from 82% to 95% (mean = 88.7 ± 5.4%). The reported AUCs ranged from 0.74 to 0.97 across eligible studies (*n* = 10), with a mean of 0.86 ± 0.08. In the subset of studies that provided such benchmarks, AI-driven models generally reported higher performance than clinicians or conventional comparator methods (e.g., 91.4% vs. 82.8% in Bowd et al. [27]). The AI-driven models evaluated in Hussain et al. [19] and Mohammadzadeh et al. [29] demonstrated notable predictive power for earlier detection of glaucoma progression, with the former achieving predictive capability up to 9 months in advance.

Despite strong individual performances, heterogeneity in reported endpoints (e.g., structural vs. functional glaucoma progression) and lack of standardized validation methods hindered direct meta-analytic synthesis. It should also be acknowledged that the included studies operationalized “progression” in different ways. While all studies were required to address glaucoma progression, some did so directly through structural change on serial OCT, whereas others approached it through prediction of future visual field deterioration or used diagnostic classification of glaucomatous damage as a proxy for progression-related change. This range reflects the current state of the field, in which detection and prediction of progression are closely intertwined, but it means that the term “detecting and predicting glaucoma progression” in the title encompasses several related, rather than identical, outcome definitions. Notably, several studies benchmarked their AI-driven models against algorithmic labels rather than clinician-verified ground truth, limiting external comparability. Overall, the evidence supports the potential of AI-driven models to augment glaucoma progression assessment, although real-world clinical integration remains dependent on transparent validation and multicenter external datasets.

### 3.4. Time-to-Detection and Early-Detection Improvement

Temporal predictive capability was variably reported (Table 3). Hussain et al. provided the most explicit temporal data, demonstrating that their multimodal DL model detected glaucoma progression 6–9 months earlier than the conventional 12-month follow-up [19]. In relative terms, this represents an approximate 50–75% earlier identification of progression in the same patient cohort. Mohammadzadeh et al. reported a mean time to glaucoma progression of 6.8 ± 4.2 years in progressing eyes, though the model’s lead time advantage was not quantified [29]. Bowd et al. assessed the performance of their AI-driven model at 12- and 24-month intervals, showing greater sensitivity at 24 months (76% vs. 50%), consistent with enhanced longitudinal signal acquisition [27].

No other studies provided explicit temporal or rate-based comparisons. Collectively, these findings highlight the promise of AI-driven models for earlier glaucoma detection, but they also highlight the need for standardized longitudinal metrics (e.g., months-to-progression and time-dependent AUC) to quantify lead-time benefits in future studies.

## 4. Discussion

In this systematic review of 13 studies evaluating the potential of AI-driven OCT-based models for detecting and predicting glaucoma progression, these models generally reported high diagnostic performance, with mean sensitivities and specificities exceeding 80% and AUC generally above 0.85. In the subset of studies that included a comparator, AI models reported higher performance than conventional methods, and a single study reported detection of glaucoma progression up to 9 months earlier than standard clinical follow-up; these comparisons should be interpreted cautiously, as most were derived from internally validated, often retrospective single-center datasets, and several were benchmarked against algorithm-derived rather than independent clinical labels. The rationale for synthesizing this evidence lies in the growing clinical burden of glaucoma, the limitations of subjective or intermittently obtained structural and functional assessments, and the increasing availability of large longitudinal imaging datasets suitable for ML-based analysis. Our findings highlight that AI algorithms have matured from early traditional classifiers to advanced DL architectures capable of integrating multimodal data and modeling temporal change, positioning these tools as promising adjuncts for earlier, more objective, and more reproducible detection of glaucoma progression.

Comparing our findings with the existing literature, there is a clear consistency in how AI-driven models have been used to support glaucoma detection and assessment of glaucoma progression. Earlier studies, such as those by Lee et al. [15] and Christopher et al. [30], showed that ML models achieved notable diagnostic performance using OCT-derived parameters. These findings align with our systematic review, in which sensitivity ranged from 66% to 100%, specificity ranged from 82% to 95%, and AUCs ranged from 0.74 to 0.97 across the included studies.

More recent studies, including Hussain et al. [19], Hou et al. [21], Huang et al. [17], Mandal et al. [18], and Mohammadzadeh et al. [29], used deeper, more advanced architectures, such as CNN-LSTM models and transformer-based networks. This evolution was also reflected in our systematic review, where studies such as Hou et al. [21] and Huang et al. [17] utilized longitudinal OCT data or self-supervised transformer models and reported some of the highest AUCs among the included studies. Nonetheless, our systematic review demonstrates that OCT remains the dominant input imaging modality for AI-based glaucoma analyses.

The OCT inputs used across the included studies can be broadly grouped into four categories: RNFL-based structural measurements, including circumpapillary RNFL thickness profiles (for example, Mariottoni et al. [16] and Nouri-Mahdavi et al. [28]); map-based OCT representations, including wide-angle, RNFL thickness, or macular thickness maps incorporating RNFL and/or ganglion cell–inner plexiform layer measurements (for example, Christopher et al. [30], Bowd et al. [27], and Pham et al. [32]); direct OCT B-scan inputs analyzed without prior segmentation (for example, Huang et al. [17]); and longitudinal time-series OCT, sometimes combined with visual field and intraocular pressure inputs (for example, Hussain et al. [19], Hou et al. [21], and Mandal et al. [18]). Cutting across these categories, the highest reported AUCs in our synthesis came from models that leveraged longitudinal information, paired structural inputs, or direct B-scan analysis rather than relying only on single-visit structural measurements, including Hou et al. (AUC 0.97 against algorithm-derived M6 labels) [21], Mariottoni et al. (AUC 0.938 from paired RNFL thickness profiles) [16], and Huang et al. (AUC 0.90 from macular B-scan analysis) [17]. This pattern is clinically coherent, because glaucomatous progression is inherently longitudinal, and models that incorporate change over time or more information-rich structural inputs may be better able to distinguish true progression from measurement variability than models based on a single scan.

Our findings are consistent with, but narrower than, the recent meta-analysis by Ling et al. (2025) [22], which pooled 48 studies across fundus photography and OCT modalities for both glaucoma diagnosis and progression prediction. That analysis reported pooled AUROCs of 0.90 (95% CI: 0.88–0.92) for fundus-based and 0.86 (95% CI: 0.83–0.90) for OCT-based diagnosis, with less robust performance for progression prediction and higher accuracy on internal compared with external validation datasets, patterns that are echoed in our descriptive synthesis. By restricting inclusion to OCT-based AI models evaluated for detecting and predicting glaucoma progression, the present review complements that broader work by providing a more granular view of OCT input types, longitudinal modeling strategies, and the persistent external validation gap relevant to OCT-based progression monitoring [22].

Studies assessing AI-driven models for glaucoma have primarily relied on internal validation. For instance, studies such as Lee et al. [15], Christopher et al. [30], Luo et al. [20], Hussain et al. [19], Tarcoveanu et al. [31], and Nouri-Mahdavi et al. [28] relied solely on internal validation, without external datasets, which limits the applicability of their findings to other clinical settings. In contrast, only one included study by Mohammadzadeh et al. [29] performed true external validation using a separate OCT dataset. Overall, the trends reported in the existing literature align with the performance patterns, validation approaches, and methodological limitations identified in the studies included in our systematic review. Several concrete obstacles explain why external validation remains scarce in this field. OCT data are highly device-dependent: segmentation algorithms, normative databases, and scan protocols differ across Cirrus, Spectralis, and swept-source platforms, so a model trained on one device often fails to transfer to another without recalibration. Reference standards for “progression” are themselves inconsistent, ranging from event-based and trend-based perimetry to structural or algorithm-derived labels, which makes a shared external benchmark difficult to assemble. Large longitudinal OCT datasets with harmonized labels are rarely shared openly because of patient-privacy constraints and institutional data-governance barriers. Potential solutions that have been proposed include multicenter consortium datasets with standardized acquisition and labeling protocols, device-agnostic preprocessing and domain-adaptation or transfer-learning methods to mitigate cross-platform variability, federated learning that allows model training across institutions without transferring identifiable data, and the use of public reference datasets to enable head-to-head benchmarking. Adoption of such approaches would allow the external, prospective validation that is currently the principal barrier to clinical translation.

Our findings have several potential implications for clinical practice. Firstly, the high diagnostic performance reported across the included studies suggests that AI-driven models can assist clinicians in evaluating structural changes related to glaucoma. Secondly, studies using longitudinal OCT data, such as Hussain et al. [19], Hou et al. [21], and Mandal et al. [18], demonstrate how AI-driven models can support clinical decision-making by providing data that shows continuous structural changes to the optic nerve and RNFL, rather than isolated single-visit measurements. Thirdly, Hussain et al. [19] reported earlier detection of glaucoma progression by approximately 6–9 months, suggesting that AI-driven models may help identify glaucoma progression earlier than conventional methods.

Most of the included studies relied on OCT-derived parameters, aligning with current clinical practice, in which OCT is widely used to assess glaucoma. As noted above, the limited use of external, multicenter validation remains the principal barrier to routine clinical adoption.

Glaucoma progression is traditionally monitored using standard automated perimetry (SAP) and OCT, supported by longitudinal clinical evaluation. Although these modalities represent the current gold standard, they are limited by variability, noise, and inconsistencies in the structure–function relationship. In a seminal review, Malik et al. [33] demonstrated that structural and functional progression often show poor concordance, noting that in large clinical trials, the first detectable change was structural in 35–55% of eyes and functional in 35–86%, depending on the cohort and the criteria applied. This dissociation, driven by measurement variability, non-neural RNFL components, test–retest variability, and differences in dynamic range, contributes to delayed identification of true glaucoma progression in routine clinical practice.

Raza and Hood [34] further highlighted that OCT-derived structural changes do not consistently precede VF deterioration, showing that both structural and functional testing are impacted by significant noise, dynamic range limitations, and residual non-neuronal tissue within OCT layers. These issues reduce sensitivity for early detection of glaucoma progression and require multiple visits and repeated testing to differentiate true change from randomness. Additionally, Weinreb et al. [35] emphasized broader challenges inherent to glaucoma monitoring, including the subjective nature of perimeter and segmentation artifacts in OCT imaging, further highlighting the limitations of current gold-standard tools.

In contrast, the AI-driven models reviewed herein demonstrated higher accuracy, greater reproducibility, and, in certain cases, earlier detection of glaucoma progression than conventional OCT and SAP methods. Bowd et al. [27] showed that a DL-based model applied to OCT imaging achieved an accuracy of 91.4%, outperforming conventional RNFL-based assessments (82.8%). Hussain et al. [19] reported that a multimodal DL model identified glaucoma progression 6–9 months earlier than the standard 12-month follow-up interval, a substantial improvement in lead time. By integrating longitudinal multimodal data and minimizing human-dependent variability, AI-driven models can detect subtle structural or functional patterns that are not apparent with conventional methods. Nevertheless, AI-driven models should be considered complementary rather than a replacement for current clinical standards. OCT and SAP remain essential for contextualizing glaucoma status, clinical decision-making, and identifying atypical features. However, given the inherent limitations of gold-standard testing, as demonstrated by Malik et al. [33] and Raza and Hood [34], integrating AI-driven models offers a promising strategy to enhance sensitivity, reduce diagnostic delays, and improve objectivity in glaucoma progression assessment.

From a clinical implementation perspective, OCT-based AI models are unlikely to serve as stand-alone decision systems in glaucoma care and are more plausibly positioned as adjunctive tools. In the near term, their most realistic role may be to triage serial OCT examinations by flagging eyes with possible subtle structural change for earlier clinician review, helping prioritize follow-up among suspected progressors, and potentially reducing delay between detection of progression and treatment escalation. Because OCT acquisition and longitudinal assessment are already embedded in routine glaucoma care, implementation at this level may require incremental workflow adaptation rather than major service redesign. The main barrier to translation is therefore less likely to be workflow integration than external validity. In our review, 12 of the 13 included studies used internal validation only, and most were derived from single-center cohorts, raising concern that device-, population-, and protocol-specific biases may limit generalizability and hinder safe clinical deployment.

Our findings suggest several directions for future research. Firstly, the ability of AI-driven models to detect glaucoma progression earlier than standard methods suggests they may capture subtle retinal changes that fall below conventional OCT detection thresholds. These micro-level patterns could represent early biological signals of neurodegeneration. Secondly, the strong performance of AI-driven models that utilize longitudinal data supports the notion that measurement trajectory rather than isolated measurements best capture glaucoma progression. Thus, AI-driven models may help identify distinct progression patterns, or “phenotypes,” enabling more individualized risk profiling. Thirdly, the superior accuracy of multimodal models suggests that glaucoma progression is inherently multifactorial and that integrating structural, functional, and clinical variables provides a more complete picture of disease behavior. Finally, variability in external performance across studies highlights the need for improved generalizability. Thus, effective clinical deployment of AI-driven models will require harmonized imaging inputs, standardized progression definitions, and robust cross-center validation.

This systematic review had several strengths. Firstly, it provides a comprehensive synthesis of AI-driven models for detecting glaucoma progression from OCT imaging, including traditional ML, DL, and transformer architectures, and offers meaningful comparisons across diverse models. Secondly, its methodological rigor was further strengthened by structured data extraction by two independent reviewers. However, the generalizability of the findings remains limited due to the predominance of single-center datasets and reliance on internal validation.

Several methodological considerations of the review process should also be acknowledged. The literature search was restricted to three databases (PubMed, Ovid, and Google Scholar) and to English-language publications, and did not include Embase, Web of Science, Scopus, or the Cochrane Library; relevant studies indexed only in those resources, or reported in specialist ophthalmology and engineering venues—such as Ophthalmology, Ophthalmology Glaucoma, the American Journal of Ophthalmology, Investigative Ophthalmology & Visual Science, Translational Vision Science & Technology, npj Digital Medicine, and IEEE Transactions on Medical Imaging—may therefore have been missed. We note, however, that in PubMed and Google Scholar index, the great majority of these journals and the principal OCT-based progression studies published in them during the search window were captured in the present synthesis. Future reviews would nonetheless benefit from a broader multi-database strategy with formal peer-reviewed search filters to ensure exhaustive coverage. The heterogeneity in study designs, AI architectures, and outcome definitions precluded formal meta-analysis, and the findings were therefore synthesized descriptively. Despite these considerations, the structured multi-reviewer screening and extraction process, prospective protocol registration, and systematic quality assessment using QUADAS-2 strengthen the reliability of the reported findings.

Our findings highlight the promising capabilities of various AI-driven models for detecting glaucoma progression using OCT imaging, which in the available studies performed comparably to, and in some reports better than, clinician or conventional assessment. These results are encouraging but remain preliminary: because most studies relied on internal validation and retrospective single-center data, the evidence is not yet sufficient to establish that these models match or exceed clinician performance in routine practice. Most of the included studies reported high AUCs, with some showing evidence of earlier detection of glaucoma progression. These findings highlight the potential for AI augmentation in clinical practice, especially in routine glaucoma surveillance. This integration will help reduce clinical workload and improve accessibility, particularly in resource-limited settings.

Future research must address the current evidence gaps identified in this review. Priority should be given to multicenter studies that include large, geographically and demographically diverse populations to improve the generalizability, enabling better evaluation of clinical performance and potential benefits for patient care. Moreover, future research should emphasize the use of external validation to enhance model reliability and applicability. These efforts will help enhance AI-based detection of glaucoma progression and broaden its applicability in routine clinical practice.

## 5. Conclusions

By restricting inclusion to OCT-based artificial intelligence models evaluated for detecting and predicting glaucoma progression, this review helps clarify three patterns that may be less visible in broader analyses. First, several of the strongest-performing approaches in our synthesis used longitudinal OCT information, paired or direct OCT structural inputs, or OCT combined with functional or clinical variables, suggesting that modeling change over time and incorporating richer structural or multimodal information may be important for capturing the progression signal. Second, external validation remains scarce across the included literature, and the resulting uncertainty regarding cross-center and cross-device generalizability represents a major limitation of the current evidence base. Third, the most plausible near-term clinical role for these models is adjunctive, supporting the triage of serial OCT examinations and earlier identification of suspected progressors rather than autonomous decision-making.

This systematic review evaluated the diagnostic and predictive performance of AI-driven OCT-based models for detecting and predicting glaucoma progression and contextualized their performance against the recognized limitations of standard automated perimetry and conventional OCT trend analysis. It also provided an overview of AI-driven models for detecting glaucoma progression from OCT images, covering traditional machine learning, deep learning, and transformer architectures, enabling meaningful comparisons among various AI-driven models. The findings demonstrate that AI-driven models show strong diagnostic accuracy in detecting and predicting glaucoma progression, with sensitivities and specificities exceeding 80%, and AUC values generally above 0.85. AI-driven models were reported to identify glaucoma progression as early as 9 months before standard methods. Furthermore, AI-driven models can integrate multimodal data to model and detect subtle structural or functional changes that are not apparent with conventional models, highlighting their potential to complement current clinical practice.

The findings propose several directions for future research, which should prioritize multicenter studies with large, diverse geographic and demographic groups to improve generalizability. Moreover, external validation is necessary to improve the reliability and applicability of AI-driven models in clinical practice.

## Figures and Tables

**Table 1:** The characteristics of the included studies.

Study (Year)	Study Design	Country/ Institution	Population (Glaucoma Type)	Mean Age (Years)	DL/ML Algorithm	Testing Dataset (Eyes)	Validation Type	Data Type
Lee et al. (2019) [15]	Cross-sectional	South Korea (Seoul Natl Univ Hosp/College of Medicine)	Young myopic normal-tension glaucoma	Progressors: 31.5 ± 7.7, non-progressors: 33.2 ± 4.3	RF and extra trees	45 eyes (21 progressors, 24 non-progressors)	Internal (3-fold CV + 70:30 split)	OCT (mGCIPL, cpRNFL) + VF + IOP + demographics
Christopher et al. (2018) [30]	Retrospective longitudinal analysis	USA (UCSD)	Primary open-angle glaucoma	69.6	PCA on SS-OCT RNFL maps + logistic regression	179 eyes (93 patients)	Internal (leave-one-out bootstrap AUC)	Wide-angle SS-OCT cpRNFL maps + SD-OCT
Luo et al. (2023) [20]	Retrospective observational	USA (Harvard)	Glaucoma (type not specified)	Median: 64.5 (54.3–72.8)	Pseudo-supervised multimodal network	1547 test samples	Internal (training/validation/testing split)	OCT RNFLT maps + Humphrey 24-2 VF vectors
Hussain et al. (2023) [19]	Longitudinal cohort	Lithuania (Vilnius Univ Hosp Santaros Klinikos)	Primary or secondary glaucoma post-trabeculectomy	67.3 ± 8.9	CNN (ResNet-34) + LSTM + pix2pix GAN	86 eyes	Internal (5-fold CV + hold-out)	SD-OCT ONH B-scans, VF, IOP
Tarcoveanu et al. (2022) [31]	Retrospective cohort	UK and Romania (Countess of Chester/St. Spiridon Iași)	POAG or ocular hypertension ± diabetes	64	RF, SVM, decision tree, *k*NN, MLP	150 visits (50 patients)	Internal (10-fold CV)	OCT RNFL + macular thickness + VF (VFI, MD, PSD)
Nouri-Mahdavi et al. (2021) [28]	Prospective cohort	USA (UCLA)	Glaucoma (type not specified)	67.3 ± 8.2	Naïve Bayes (best) + RF, SVM	104 eyes (104 patients)	Internal (5-fold CV)	SD-OCT (cpRNFL + macular GCIPL)
Mariottoni et al. (2023) [16]	Retrospective cohort	USA and Brazil (Duke/São Paulo)	Glaucoma, suspects, and healthy controls	64.5 ± 12.6	Custom CNN	816 eyes (462 individuals)	Internal (5-fold CV, patient-stratified)	SD-OCT RNFL thickness
Hou et al. (2023) [21]	Retrospective longitudinal cohort	USA (Johns Hopkins and Iowa)	Glaucoma suspects and Glaucoma patients	64.7 ± 11.8	GTN	4211 eyes (2666 patients)	Internal (65/17.5/17.5 split)	HD-OCT RNFL
Mohammadzadeh et al. (2024) [29]	Retrospective observational cohort	USA (UCLA Stein Eye Institute)	POAG, NTG, pigmentary, pseudoexfoliative, and angle-closure glaucoma	Stable: 59.7, Progressing: 61.6	CNN (ResNet-152, ImageNet pretrained)	3079 eyes (1765 patients) + 427 external eyes	Internal + external (80/10/10 split + OCT external set)	Fundus optic disk photos + OCT RNFL
Huang et al. (2024) [17]	Retrospective cohort	USA (Northwestern Univ.)	Primary open-angle glaucoma	Detection: 67.4–70.0, Prediction: 66.5–67.7	ViT (DINO self-supervised)	Detection: 307 eyes, Prediction: 241 eyes	Internal (temporal hold-out + 80/20 split)	macular OCT B-scans
Bowd et al. (2021) [27]	Prospective cohort	USA (UCSD + ADAGES)	Open-angle glaucoma (progressing and non-progressing)	Healthy: 54.3, non-progressing: 71.7, Progressing: 61.4	Unsupervised auto-encoder + ROI/Markov segmentation	237 eyes (153 patients)	Internal (DIGS/ADAGES cross-validation)	SD-OCT RNFL
Pham et al. (2024) [32]	Retrospective observational	USA (Johns Hopkins)	Glaucoma and glaucoma suspects	64 ± 14	RF, SVM, *k*NN, naïve Bayes	617 test eyes (1541 total eyes)	Internal (60/40 split + 5-fold CV)	SD-OCT (cpRNFL + GCIPL)
Mandal et al. (2024) [18]	Retrospective cohort	USA (Bascom Palmer and Duke)	Open-angle glaucoma and healthy controls	65.6 ± 10.5 (overall)	Noise positive-unlabeled time-series CNN-LSTM (ResNet-50 + 3D-CNN front-end)	1225 test sequences (3253 total eyes)	Internal (70/15/15 subject-level split)	SD-OCT time-series

Abbreviations: ADAGES, African Descent and Glaucoma Evaluation Study; AUC, area under the receiver operating characteristic curve; CNN, convolutional neural network; cpRNFL, circumpapillary retinal nerve fiber layer; CV, cross-validation; DIGS, Diagnostic Innovations in Glaucoma Study; GAN, generative adversarial network; GCIPL, ganglion cell–inner plexiform layer; GTN, gated transformer network; HD-OCT, high-definition optical coherence tomography; IOP, intraocular pressure; *k*NN, *k*-nearest neighbors; LSTM, long short-term memory; MD, mean deviation; mGCIPL, macular ganglion cell–inner plexiform layer; MLP, multilayer perceptron; NTG, normal tension glaucoma; OCT, optical coherence tomography; PCA, principal components analysis; POAG, primary open-angle glaucoma; PSD, pattern standard deviation; ResNet-34, 34-layer residual network; ResNet-50, 50-layer residual network; ResNet-152, 152-layer residual network; RF, random forest; RNFL, retinal nerve fiber layer; ROI, region of interest; SD-OCT, spectral-domain optical coherence tomography; SS-OCT, swept-source optical coherence tomography; SVM, support vector machine; UCLA, University of California–Los Angeles; UCSD, University of California–San Diego; VF, visual field; VFI, visual field index; ViT, vision transformer.

**Table 2:** Comparison of the diagnostic performance of AI-driven models and clinicians.

Study (Year)	Sensitivity (%)		Specificity (%)		AUC (95% CI, Where Available)	Accuracy (%)	
	AI	Clinicians	AI	Clinicians		AI	Clinicians
Lee et al. (2019) [15]	83.7	N/A	85.1	N/A	0.881	79.8	N/A
Christopher et al. (2018) [30]	N/A	N/A	N/A	N/A	0.74 (95% CI: 0.62–0.85) for mean cpRNFLt; 0.74 (95% CI: 0.61–0.87) for SAP 24–2 MD; 0.71 (95% CI: 0.56–0.86) for FDT MD	N/A	N/A
Luo et al. (2023) [20]	N/A	N/A	N/A	N/A	Unimodal TD: 0.7447 ± 0.0084, MD Fast: 0.7004 ± 0.0218, Pseudo-Sup + Aug TD: 0.7477 ± 0.0074, Multimodal TD: 0.7517 ± 0.0041	82.24	N/A
Hussain et al. (2023) [19]	N/A	N/A	N/A	N/A	0.83 (6 months earlier), 0.81 (9 months earlier with synthetic OCT)	N/A	N/A
Tarcoveanu et al. (2022) [31]	76.47	N/A	95.18	N/A	N/A	N/A	N/A
Nouri-Mahdavi et al. (2021) [28]	66	N/A	85	N/A	0.81	N/A	N/A
Mariottoni et al. (2023) [16]	87.3	N/A	86.4	N/A	0.938	N/A	N/A
Hou et al. (2023) [21]	100 (per M6 labels)	N/A	93 (per M6 labels)	N/A	0.97 (per M6 labels)	N/A	N/A
Mohammadzadeh et al. (2024) [29]	81.90	N/A	85.8	N/A	0.894	85	N/A
Huang et al. (2024) [17]	84	N/A	82	N/A	0.90 (detection), 0.85 (prediction)	N/A	N/A
Bowd et al. (2021) [27]—12 months	50	22 (comparator cpRNFL annulus)	70	68 (comparator)	N/A	91.41	82.81
Bowd et al. (2021) [27]—24 months	76	51 (comparator)	83	81 (comparator)	N/A	N/A	N/A
Pham et al. (2024) [32]	N/A	N/A	N/A	N/A	RNFL: 0.78, GCIPL: 0.75, Combined 0.81	N/A	N/A
Mandal et al. (2024) [18]	Hit ratio: 49.8 (uncertain equivalence to sensitivity)	N/A	95	N/A	N/A	N/A	N/A

Notes: For Tarcoveanu et al. (2022), values were calculated from the reported confusion matrix (true positive = 13, false negative = 4, true negative = 79, false positive = 4) [31]. For Bowd et al. (2021), accuracy values were recomputed from the provided true-positive and true-negative counts [27]. For Hou et al. (2023), results labeled “per M6 labels” indicate evaluation against algorithm-derived labels rather than independent clinical adjudication [21]. Abbreviations: AUC, area under the receiver operating characteristic curve; CI: confidence interval; GCIPL, ganglion cell–inner plexiform layer; MD, mean deviation; N/A, not reported; RNFL, retinal nerve fiber layer; TD, total deviation.

**Table 3:** Improvement in time-to-detection and early-detection of glaucoma progression.

Study (Year)	Time-to-Detection	Early Detection Improvement
Hussain et al. (2023) [19]	6–9 months earlier than the 12-month standard	Approx. 50–75% earlier detection
Mohammadzadeh et al. (2024) [29]	Mean 6.8 ± 4.2 years to progression	Not quantified
Bowd et al. (2021) [27]	12 vs. 24 months	Sensitivity improved from 50% to 76%

## Data Availability

The original contributions presented in this study are included in the article/Supplementary Materials. Further inquiries can be directed to the corresponding author.

## Supplementary Materials

The following supporting information can be downloaded at: https://www.mdpi.com/article/10.3390/jcm15155976/s1. PRISMA 2020 checklist.

## Abbreviations

The following abbreviations are used in this manuscript:

ADAGES	African Descent and Glaucoma Evaluation Study
AI	Artificial Intelligence
AUC	Area Under the Receiver Operating Characteristic Curve
CI	Confidence Interval
CNN	Convolutional Neural Network
cpRNFL	Circumpapillary Retinal Nerve Fiber Layer
CV	Cross-Validation
DIGS	Diagnostic Innovations in Glaucoma Study
DL	Deep Learning
GAN	Generative Adversarial Network
GCIPL	Ganglion Cell–Inner Plexiform Layer
GTN	Gated Transformer Network
HD-OCT	High-Definition Optical Coherence Tomography
IOP	Intraocular Pressure
kNN	k-Nearest Neighbors
LSTM	Long Short-Term Memory
MD	Mean Deviation
mGCIPL	Macular Ganglion Cell–Inner Plexiform Layer
ML	Machine Learning
MLP	Multilayer Perceptron
N/A	Not Reported
NTG	Normal Tension Glaucoma
OCT	Optical Coherence Tomography
PCA	Principal Component Analysis
POAG	Primary Open-Angle Glaucoma
PRISMA	Preferred Reporting Items for Systematic Reviews and Meta-Analyses
PSD	Pattern Standard Deviation
QUADAS-2	Quality Assessment of Diagnostic Accuracy Studies-2
ResNet-34	34-Layer Residual Network
ResNet-50	50-Layer Residual Network
ResNet-152	152-Layer Residual Network
RF	Random Forest
RNFL	Retinal Nerve Fiber Layer
ROI	Region of Interest
SAP	Standard Automated Perimetry
SD	Standard Deviation
SD-OCT	Spectral-Domain Optical Coherence Tomography
SS-OCT	Swept-Source Optical Coherence Tomography
SVM	Support Vector Machine
TD	Total Deviation
UCLA	University of California–Los Angeles
UCSD	University of California–San Diego
VF	Visual Field
VFI	Visual Field Index
ViT	Vision Transformer
